# Prevalence of Upper Extremity Musculoskeletal Disorders in Dentists: Symptoms and Risk Factors

**DOI:** 10.1155/2015/517346

**Published:** 2015-05-03

**Authors:** Forouzan Rafie, Azadeh Zamani Jam, Arash Shahravan, Maryam Raoof, Ali Eskandarizadeh

**Affiliations:** ^1^Oral and Dental Diseases Research Center, Kerman University of Medical Sciences, Kerman, Iran; ^2^Tehran University, Iran; ^3^Laboratory of Molecular Neuroscience, Neuroscience Research Center, Institute of Neuropharmacology, Kerman University of Medical Sciences, Kerman, Iran

## Abstract

*Aim*. The purpose of the present research was to examine the factors that lead to musculoskeletal disorders in dentists by assessing their posture using RULA method. *Materials and Methods*. In this cross-sectional study, 130 dentists (84 male and 46 female) participated. The posture of the subjects during their normal workload was recorded by using the RULA method, and the range of musculoskeletal pains by using the Nordic Musculoskeletal Questionnaire (NMQ), and individual and professional data was assessed by a demographics questionnaire. All tests were performed at the *P* < 0.05 level. *Results*. Assessment of the physical status of the subjects showed that 82.8% of subjects were at high risk of musculoskeletal disorders. The majority of musculoskeletal pains were in the neck (55.9%) and the shoulder (43.8%). Moreover, 68.9% of the subjects had experienced pain at least once over the last year. Significant relationships were observed between musculoskeletal pain and daily work hours (*P* = 0.07) and number of patients (*P* = 0.02), but the pain was not significantly associated with BMI and experience. *Conclusion*. The present findings showed that unsuitable posture of dentists during work has a considerable effect on musculoskeletal disorders. Therefore, further investigation is required to avoid the detrimental effects of wrong posture.

## 1. Introduction

Dental practice is a high risk profession and every year a large number of dentists are at risk of job related musculoskeletal disorders [1–3]. Different factors such as heredity, stress, unsuitable posture during work, and lack of regular exercise can affect the incidence of such disorders [3]. In many studies of the prevalence of different musculoskeletal pains, it was reported as more than 50% [4–6], but due to poor posture of upper limb [7, 8], repetitive movements, long term static contractions [9], and use of high-vibration tools [10], accumulation and injury in the upper limb are higher [11, 12]. A review by Hayes et al. (2009) reported the majority of damage in upper limbs (back, shoulder, and neck) [13]. In the study of Chamani et al., the prevalence of pain in dentists over a year was reported to be 50.9%, 43.6%, and 37.3% in the neck, wrist, and back regions, respectively [14]. Many studies have reported that poor posture in dentists during working is the major risk factor for musculoskeletal pain; for example, Choobineh and his colleagues, in Shiraz (2010), reported that dentists' postures during dental work, by RULA (Rapid Upper Limb Assessment) method, were at high risk of musculoskeletal injuries and require immediate attention and correction [15]. In similar studies, Saremi in Tehran (2006) [16] and Varmazyar et al. in Gazvin (2008) [17], respectively, reported that 30% and 30.2% of dentists were prone to the risk of musculoskeletal disorders by using REBA (Rapid Entire Body Assessment) method. A study by the American Dental Association (1997) showed that 9.2% of the subjects had serious upper limb damage of which 20% required surgery and 40% had to reduce their work hours [18]. Shugars et al. (1984) reported that annually more than 41 million dollars is spent on treatment of musculoskeletal disorders in dental professionals [19]. Each year many dentists are forced to leave their clinic, reduce their work hours, and even consider early retirement [19–21]. High treatment costs and work loss increase economic problems and decrease efficiency.

Due to the negative effects of musculoskeletal injuries, review and study of injuries and musculoskeletal pain is very important, but since most previous research aimed to investigate the prevalence of musculoskeletal disorders [14, 21, 22] and less attention to injuries of upper limb postures and specifically to the effect of body posture, the present study aimed to assess posture versus upper limb musculoskeletal pain among dentists.

## 2. Materials and Methods

In this descriptive cross-sectional study, 130 general and professional dentists (84 male and 46 female) with at least 4 years of experience and with no record of hereditary musculoskeletal disorders participated in the sample; they used new and up-to-date equipment. Of this number, 12 subjects were excluded from the study due to their lack of cooperation or submitting incomplete questionnaires. Using a demographic questionnaire, the personal characteristics of the subjects were recorded, including gender, age, height, weight, BMI, regular exercise pattern every week, work experience, work hours per week, and number of patients per week. Moreover, the RULA (Rapid Upper Limb Assessment) method was used to examine the ergonomic sitting posture of the subjects. This method was developed by McAtamney and Corlett (1993) for use in ergonomic investigations of workplaces where work related upper limb disorders are reported [23].

This method was used several times to verify the employment status of many professions, including dentistry [15, 24, 25]. The dominant side of the body was assessed using this method. RULA examines the number of movements, static muscle work, and force as risk factors. At first, body limbs are divided into two groups: group A which includes the upper arm, lower arm, and wrist and group B which includes the neck, trunk, and legs. The range of movement for each body part is divided into different sections and each section is numbered based on its deviation from the normal posture. Accordingly, score 1 is given to the range of movement or sitting working posture where the risk factors present are minimal, and higher scores are allocated to parts of the movement range with more extreme postures indicating an increasing presence of risk factors. If the body part is deviated from the midline or rotated, the number attributed to the posture increases. Afterwards, the scores of different body parts are combined, and given the muscular activity and the exerted force, a final score is derived which indicates the level of risk of injury (Tables 2 and 3). The ergonomic posture of subjects during sitting work is examined for 20 minutes, movements of different body parts are observed and recorded, and the worst and most frequent postures are assessed.

In addition, Nordic Musculoskeletal Questionnaire (NMQ), developed by Kourinka et al. (1987), was used to examine the incidence and prevalence of musculoskeletal disorders among the subjects during the past 12 months [26]. The questionnaire is used as a tool for screening musculoskeletal disorders in epidemiological studies [14], but it cannot be used for clinical diagnosis. The questionnaire consists of two parts: (A) general questionnaire to answer the question of whether problems of musculoskeletal exist and if so where the focus is in the organs of the human body taking this issue into nine anatomical sites (neck, shoulders, back, elbows, back, wrist/hand, hip/thigh, knee, and ankle/foot) or groups and to investigate the area and its history of pain in the last 12 months or inability to leave work and the impact of these problems on the person doing deals and (B) specific questionnaire to provide a deeper analysis of these symptoms in certain areas of the body such as back, neck, and shoulders and career history and its impact on individual deals.

Individual and job characteristics and RULA scores of dentists were reported by mean, standard deviation, and frequency; for determining relation between RULA scores and symptoms of pain in the same limb, data were analyzed using logistic regression analysis; for determining relation between symptoms of pain and variables, chi-squared test was used; to compare two groups, women and men, we used *t*-test. All tests were performed at the *P* < 0.05 level.

## 3. Results

Personal characteristics and work profile of subjects are listed in Table 1. Based on data collected, 26.3% of participants exercise regularly every week (Table 1).

RULA scores are reported in Table 2; the final score obtained (6.50) was most affected by the high rating of group A (neck, shoulder, and elbow) (Table 2).

Postural assessment using RULA indicated that 57% of the subjects were at action level 3; that is, they soon required further investigation and action. Further, 24.8% of the subjects required immediate change (Table 3).

Table 4 provides the mean RULA scores based on the type of work, where surgeons and prosthodontics had the highest risk levels (Table 4). To determine the relationship between RULA scores and the pain that was reported for each part of the body, the Nordic questionnaire logistic regression was used, and predictive values (PV) and confidence intervals (CI) at 95% significance level were used. The level of significance was considered at *P* ≤ 0.05 (Table 5). The results of *t*-test between two groups, men and women, showed there was no significant difference in the final score RULA (*P* = 0.06).

Based on the data from NMQ, the highest prevalence of musculoskeletal pain during the past 12 months was related to the neck (55.9%), shoulder (43.8%), waist (39.2%), wrist (34.5%), and back (32.5%) regions (Table 6).

Based on the results presented in Table 7, frequency and prevalence of pain symptoms in women were generally more than in men, but this difference was significant only at the wrist (*P* = 0.03) (Table 7).

The results suggest that there is a significant relationship between the prevalence of musculoskeletal disorders and number of patients per week (*P* = 0.02) and work hours per week (*P* = 0.007), but it was not significantly associated with gender, experience, and BMI (*P* > 0.05) (Table 8).

Moreover, 15% of the subjects had to leave their clinic or reduce their work hours, and overall 68.9% of the subjects reported that they had experienced pain and discomfort at least once over the last year.

## 4. Discussion

The numbers of patients with musculoskeletal pain of the upper limb in dentists are growing, so that the prevalence of it from 58% in 2001 [27] has reached to 81% in 2006 [28]. Due to the increasing prevalence of musculoskeletal pain in dentists, the present research studies the prevalence and risk factors of upper limb musculoskeletal disorders in dentists.

The results of this research showed that 68.9% of the subjects reported symptoms of musculoskeletal disorders over the last year, and this prevalence of such disorders is consistent with many studies (e.g., 64% of the subjects in study of Marshall et al., 64.8% [29], in Motamayel et al. 47% [30], in the review article of Hayes et al. [13], 73% of the subjects in the study of Rabiei et al. [31], and 81.6% of the subjects in Ardakani research reported it [32]). These corresponded or differed with the prevalence and incidence of pain sometimes, probably due to differences between the individual and social participant.

The results also indicated that the region mostly associated with musculoskeletal disorders is the neck with a prevalence of 55.9%. This is consistent with the findings of Al Wazzan et al. (54.5%) [33], Al Wazzan et al. (54.4%) [34], Leggat et al. (57.5%) [2], Varmazyar et al. (50.8%) [17], and Chamani (50.9%) [14]. Prevalence of pain in the neck and shoulder is due to the nature of dental practice. Leaning forward to an angle of 15° or sometimes 30° and remaining in this posture for a long period of time along with elevating the shoulders (with more than 30° abduction or flexion) exerts much pressure on the neck and the shoulder [9, 25, 35, 36].

Prevalence of musculoskeletal disorders was generally higher in women than men, which may be associated with the lower muscle volume and strength of women and female hormones. However, except for the wrist, no significant gender differences were observed in musculoskeletal disorders. This is consistent with the results of Leggat et al. [2], Garcia et al. [25], Coury et al., Kerosuo et al., Åkesson et al., and Diaz-Caballero et al. [37–40] but inconsistent with the findings of Chamani et al. [14]. The results also suggested no significant relationship between weight, BMI, and prevalence of pain, and this is consistent with the findings of Lindfors et al. [28] and Chamani et al. [14] but inconsistent with the findings of Motamayel et al. [30].

The findings showed that prevalence of musculoskeletal pain was positively associated with work hours. Indeed prolonged static contractions lead to accumulation of lactic acid, reduction of oxygen levels, and fatigue and pain. Chubine also highlighted this issue, but this finding is inconsistent with the results of Marshall et al. [29], Chamani et al. [14], and Al Wazzan et al. [34].

In the present study, oral surgeons and prosthodontics specialists were the most vulnerable groups for musculoskeletal disorders. Also in the studies of Ratzan and Harutunian [7, 41] the highest prevalence of musculoskeletal disorders occurred in oral surgeons due to their more stressful work. Moreover, in the study of Varmazyar et al. [17] highest REBA action level belonged to prosthodontics specialists due to their static work and oral surgeons due to their stressful work that required much concentration.

There is much inconsistency regarding the effect of the years of work on the incidence and prevalence of musculoskeletal disorders. Some studies have reported that such disorders increase with the years of work [7], while others have reported higher prevalence in the unskilled [42]. In the present study, however, musculoskeletal disorders were not significantly associated with work experience. This may be due to the fact that the majority of the subjects were young and adverse working conditions and other variables had not yet affected them. This finding is consistent with the results of Chamani et al. [14], Al Wazzan et al. [34], Choobineh et al. [15], and Marshall et al. [29].

Regular aerobic and stretching exercises are a key factor in preventing damage and strengthening the musculoskeletal system in dental workers [42, 43]. Although the present study showed that musculoskeletal disorders were less prevalent in dentists who performed regular exercises, no significant relationship was observed between exercise and incidence of pain, which is consistent with the results of Pour-Abbas et al. [36] and Augustson and Morken [44].

In the present research, a significant relationship was observed between RULA scores for different regions of the body and the pain reported by the subjects. In a similar study, Seraji [21] found that the pain reported in different body parts was related to the working conditions associated with the same body parts, while in the research of Varmazyar no significant relationship was observed between REBA scores and the pain related to the same body part.

Although 68.9% of the subjects reported at least one instance of upper limb discomfort over the last year, only 29.2% of them had taken action to improve or treat their disorder. In the study of Harutunian, 39.7% of dental workers were willing to investigate further, and 66.2% had taken no action to improve their problem [41].

Reporting of the most severe pain in the neck and shoulder and the high RULA grand score (6.50) indicated that the subjects were mostly affected by the high group A score (i.e., the neck, shoulder, and wrist). Lack of control over the patient, insufficient lighting, and working in a small environment with limited access force the dentist to work in a seated and forward-leaning position for long period of time with 15–30° flexion which induces much stress and potential damage to the upper limbs of the dentists [9, 35, 45]. This finding is consistent with the results of Saremi [16], Varmazyar et al. [17], Choobineh et al. [15], and Garcia et al. [25].

The present findings showed that 15% of the subjects had to leave their clinic or reduce their work hours due to musculoskeletal disorders, and Khalid et al. (24%), Pour-Abbas (11.3%), and Saremi (17%) had reported work loss and absenteeism as a result of such disorders.

## 5. Conclusion

High frequency of pain and high risk levels, according to the RULA method, suggest inappropriate and incorrect ergonomic postural habits existing among dental professionals. Since there are not any teaching or ergonomic classes in dental universities in Iran, this study suggests an intervention program about work related musculoskeletal disorders that focuses on ergonomic considerations and regular exercises that can be effective in reducing such disorders.

## Figures and Tables

**Table 1:** Demographics of the subjects (mean ± standard deviation).

Indices	Height (m)	Weight (kg)	BMI (kg/m^2^)	Age (years)	Professional experience (years)	Work hours per week	Number of patients per week	Exercise weekly (hour)
Women	158.61 ± 1.2	65.13 ± 1.1	24.5 ± 0.2	35.1 ± 1.8	8.91 ± 3.9	37.74 ± 2.9	33.65 ± 1.6	2.55 ± 1.1
Men	174.21 ± 0.2	80.33 ± 0.5	27.8 ± 3.0	37.02 ± 2.2	11.01 ± 1.4	44.13 ± 4.6	41.27 ± 2.9	4.07 ± 0.3

Total	168.58 ± 3.71	72.35 ± 1.04	26.8 ± 1.1	36.21 ± 2.07	9.19 ± 4.31	41.02 ± 4.01	36.00 ± 2.89	3.13 ± 1.14

**Table 2:** RULA scores in groups of body limbs.

Group	RULA scores (mean ± SD)
Group A (neck, shoulder, and wrist)	7.70 ± 0.09
Group B (waist and back)	1.02 ± 5.30
Grand score	6.50 ± 1.43

**Table 3:** Final scores and action levels.

RULA score (level)	Percentage	Required action
1 & 2 (action level 1)	0%	Posture is acceptable if it is not maintained or repeated for long periods
3 & 4 (action level 2)	18.2%	Further investigation is needed and changes may be required
5 & 6 (action level 3)	57.0%	Investigation and changes are required soon
7 and above (action level 4)	24.8%	Investigation and changes are required immediately

**Table 4:** Mean RULA scores and action levels based on the type of work.

Type of work	*N*	Mean score	Action level
Surgery	14	9.2	4
Dentures	13	8.4	4
Fixed prosthodontics	14	7	3
Periodontics	17	6.8	3
Reconstructive surgery	18	6.4	3
Endodontics	14	7.5	3
Children	18	5.8	3
Orthodontics	10	5.1	3

**Table 5:** The regression results for the relationship between RULA scores and NMQ data.

Body part	OR	CI
Neck^*^	2.1	0.98–1.47
Shoulder	1.00	0.82–1.11
Wrist	0.98	0.88–1.21
Trunk	1.03	0.76–1.34

The results of *t*-test between two groups, men and women, showed there was no significant difference in the final score RULA (*P* = 0.06).

^*^significant.

**Table 6:** The relationship between reported pain and RULA scores.

Region	Percentage	Sig.	Relationship with RULA scores
Neck^*^	48.6%	0.017	Exists
Shoulder^*^	42%	0.027	Exists
Wrist	28%	0.38	Does not exist
Trunk	38.8%	0.14	Does not exist

Notes: *P* ≤ 0.05.

^*^significant.

**Table 7:** Prevalence of pain with respect to body parts and gender.

Reported region	Men	Women
Neck	54.2%	57.6%
Shoulder	42.7%	44%
Wrist	27.9%	40.2%
Back	30.6%	34.4%
Waist	38.5%	39.9%

**Table 8:** The relation between prevalence of musculoskeletal disorders and personal and occupational characteristics.

Variable	*X*2	*P* value
Gender	1.18	0.93
BMI	1.02	0.88
Experience	0.98	0.87
Number of patients per week^*^	0.02^*^	0.91
Exercise hours per week	1.56	0.21
Work hours per week^*^	0.007^*^	0.53

^*^significant.
